# Cerebro - Spinal Pneumonetis, with Articular Rheumatic Pains

**Published:** 1872-05-01

**Authors:** J. T. Everett

**Affiliations:** Sterling, Ill.


### CEREBRO-SPINAL PNEUMONETIS, WITH ARTICULAR RHEUMATIC PAINS.

BY J. T. EVERETT, A.M., M.D., STERLING, ILL.

I report the following epidemic, which has been prevailing in this vicinity for the past three months, as being unique and to me singular, it not being mentioned in any work or periodical to which I have access.

I report these cases for the purpose of learning if other practitioners have met with similar cases, and to learn what to call it.

It attacks children more frequently than adults, perhaps most frequently between the ages of three and eight years. Of fifty-three cases, only four were infants under two years, fifteen were between the ages of two and six, twenty-four between six and twelve, and eight were between the years of twelve and sixteen, while only two were adults.

The premonitory symptoms were lassitude, dull pain in the head, back and limbs, with slight cough, nausea and general malaise, accompanied by obstinate constipation.

These symptoms, with furred tongue, face slightly flushed, and increasing pain in the head, continue for from 12 to 24 hours, when the stage of active invasion is ushered in by agonizing occipital pains, wandering and disordered intellection. The concentration of thought is impossible. Severe pulmonic congestion now sets in, with dry, hacking cough, pain in the side, and high febrile excitement —pulse running from 120 to 190, according to age. The face red and suffused; tongue coated with white fur in the center, and with the edges red and granulated. The eyes are much injected—the pupil at times contracted, at others dilated; in a few cases strabismus was remarked.

After this has continued for a short time, there supervenes severe muscular rigidity of the back and neck, with spasmodic twitching of the limbs, etc.

The lung trouble now assumes the characteristic pneumonic symptoms. The sputa, which was at first white and frothy, now assumes the brick dust hue, while the rales become more coarse and cover over more space. This stage lasts from 24 to 36 hours, and sometimes two or three days, when the lung difficulty slowly subsides, the pulse lessens in frequency and in tenseness, the countenance becomes pale and pinched, the pain in the head lessens, but the articular pains augment in severity, accompanied by swelling and inflammation.

Sordes now appears on the teeth and gums, a sero-saneous discharge runs from the nose and mouth, the fur upon the tongue assumes a dark red or coppery hue, while the edges are red and soft, marked with the prints of the teeth. The intellection is still sluggish, and the occasional twitching of the muscles show marked cerebro-spinal irritation.

During all this time the bowels remain obstinately constipated, refusing to respond to ordinary laxatives. The urine is generally scanty and high colored, and heavily loaded with lithates, phosphates and epithelial casts, and in a few cases faint traces of albumen appeared upon the application of heat and nitric acid.

From this time on (in favorable cases), the patients made a slow and tedious recovery, it often taking two and even four weeks before convalescence is fully established.

Out of fifty-three cases, three proved fatal; the rest recovered without troublesome sequella.

In all three of these fatal cases, well marked cerebro-spinal meningitis set in, and in two cases the pathognomonic spots or petechiæ appeared, one case only living three hours from the active attack.

*Treatment.*—The remedies which have been most successful in my hands have consisted in anti-periodic doses of quinine in the first stages, with saline cathartics or blue mass, followed by a free use of diuretics and diaphoretics, pulv. Doveri, spts. Minderiri, etc., etc. In asthenic and obstinate cases I give iron and quinine from beginning to end of sickness. When there is much spinal irritation, I have used with much benefit the Calabar bean in connection with bromide of potassium, lithium and ammonium, chloral, gelseminum, etc. After the final irritability begins to subside, I use ergot, erigeron canadense and iodide of pot., with smaller doses of bromide and Calabar bean, and the valerianates of iron, quinia, zinc, cadmium, etc. During the stage of depression, I use strychnia and nitric acid only in cases where the spinal irritation has been not well marked, but the cerebral torpor indicates a stimulant tonic.

*Queries.*—This disease is evidently epidemic though not contagious, is probably (?) of malarial origin; but what is the cause of the intense and separate inflammation of different parts which have no connection or sympathy? Were these fifty-three cases simply cases of modified cerebro-spinal meningitis, or were they a new modification of intermittent or miasmatic fever? If the latter, I would respectfully suggest to "Dame Nature" that the catalogue of malarial troubles is quite extensive enough already, to warrant her in foregoing any further experimentation in that direction.

